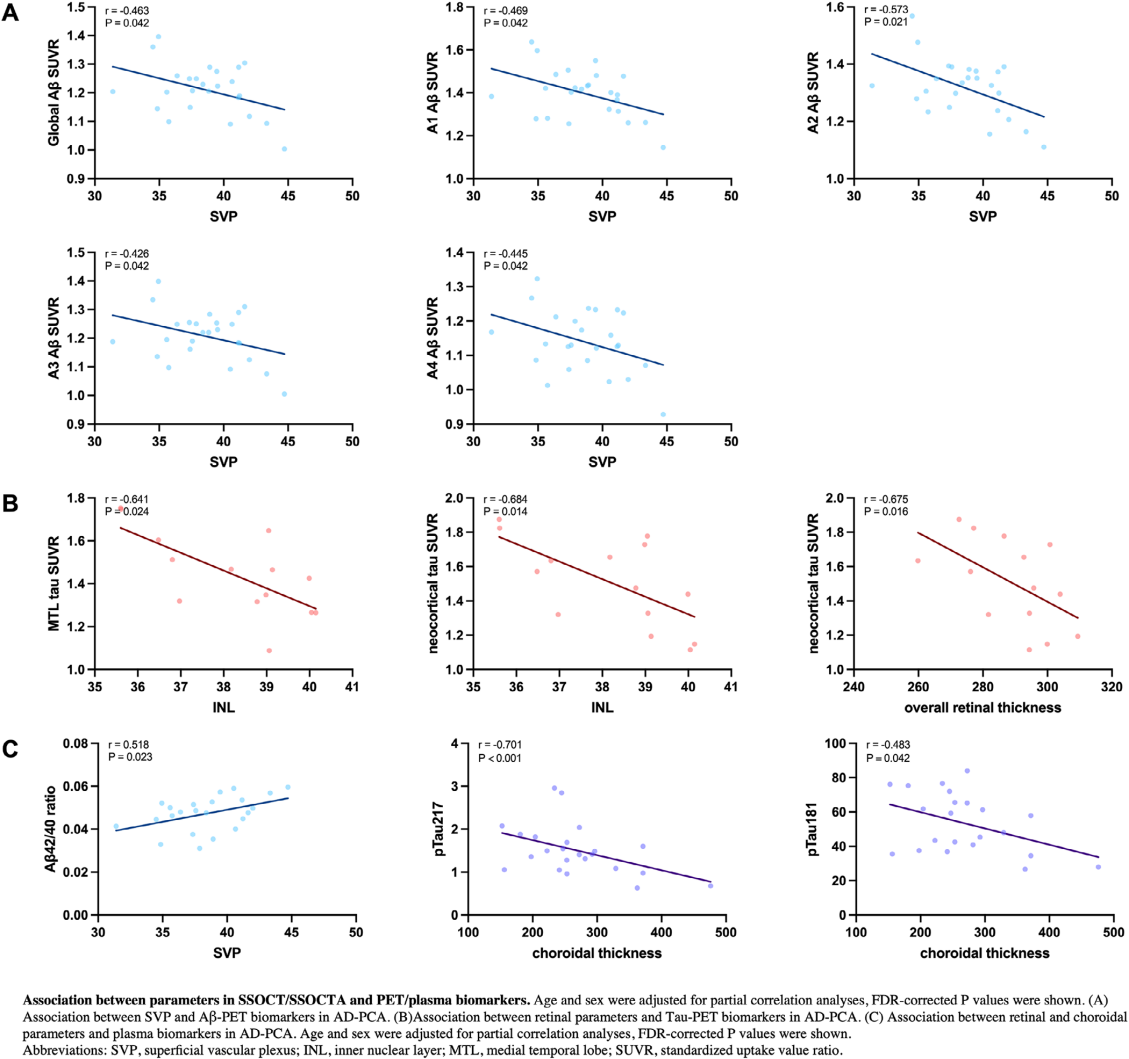# Retinal parameters in SSOCT/SSOCTA associated with PET and plasma biomarkers in Posterior Cortical Atrophy due to Alzheimer's disease

**DOI:** 10.1002/alz70856_105394

**Published:** 2026-01-08

**Authors:** Ruihan Wang, Yuzhu Gao, Li Li, Chunyan Luo, Rong Tian, Su Lv, Ming Zhang, Qin Chen

**Affiliations:** ^1^ West China Hospital of Sichuan University, Chengdu, Sichuan, China

## Abstract

**Background:**

Histopathological studies have identified Aβ and phosphorylated tau proteins in the post‐mortem retina of patients with Alzheimer's disease (AD). Retinal and choroidal alterations have been found in patients with posterior cortical atrophy (PCA) compared with healthy controls. However, the relationship between retinal and choroidal changes and in vivo AD pathology in PCA due to AD (AD‐PCA) remains poorly understood. Our aim was to explore the association between retinal parameters and the PET and plasma biomarkers in AD‐PCA.

**Method:**

This cross‐sectional study included patients diagnosed with AD‐PCA, confirmed by Aβ‐PET, from the West China Hospital (*n* = 25, mean age 60.35 ± 6.16, 32% male). Retinal and choroidal structural and microvascular parameters were assessed using swept‐source optical coherence tomography (SS‐OCT) and angiography (SS‐OCTA). Volume‐weighted global Aβ SUVR and four meta‐ROIs SUVRs corresponding to the stages of Aβ deposition from early (A1) to late (A4), were calculated to quantify Aβ burden in Aβ‐PET. In a subcohort who also received tau‐PET (*n* = 14), volume‐weighted tau SUVR values were quantified in the medial temporal lobe (MTL) and neocortex (representing Braak stages V and VI). Partial correlation analyses were performed to assess correlations between retinal parameters, and Aβ‐PET, tau‐PET or plasma biomarkers (Aβ42, Aβ40, pTau181, and pTau217) adjusting for sex and age. False discovery rate (FDR) method was applied for multiple comparisons.

**Result:**

The vessel density of the superficial vascular plexus (SVP) was inversely correlated with both global Aβ burden (r=‐0.463, *p* = 0.042) and Aβ burden across stages (A1, r=‐0.469, *p* = 0.042; A2, r=0.573, *p* = ‐0.021; A3, r=‐0.426, *p* = 0.042; A4, r=‐0.445, *p* = 0.042). Lower thickness of the retinal inner nuclear layer was associated with higher tau burden in both the MTL (r=‐0.641, *p* = 0.024) and neocortex (r=‐0.684, *p* = 0.014). While overall retinal thickness was negatively correlated only with the neocortical tau SUVR (r=‐0.675, *p* =  0.016). Additionally, SVP vessel density was positively correlated with the plasma Aβ42/40 ratio (r=0.518, *p* = 0.023). Decreased choroidal thickness was associated with higher levels of both plasma pTau217 (r=‐0.701, *p* <0.001) and pTau181 (r=‐0.483, *p* = 0.042).

**Conclusion:**

Retinal structural and microvascular biomarkers may provide valuable insights into early amyloid and tau deposition, as well as the biological progression of AD‐PCA.